# Characterization of a Novel Mitovirus Infecting *Melanconiella theae* Isolated From Tea Plants

**DOI:** 10.3389/fmicb.2021.757556

**Published:** 2021-11-17

**Authors:** Karim Shafik, Muhammad Umer, Huafeng You, Hamdy Aboushedida, Zhenhua Wang, Dejiang Ni, Wenxing Xu

**Affiliations:** ^1^Hubei Hongshan Laboratory, Huazhong Agricultural University, Wuhan, China; ^2^Department of Plant Pathology, Faculty of Agriculture, Alexandria University, Alexandria, Egypt; ^3^Key Laboratory of Horticultural Plant Biology, College of Horticulture and Forestry Sciences, Ministry of Education, Huazhong Agricultural University, Wuhan, China; ^4^Key Lab of Plant Pathology of Hubei Province, Wuhan, China; ^5^College of Plant Science and Technology, Huazhong Agricultural University, Wuhan, China; ^6^Technology Center of Wuhan Customs District, Wuhan, China

**Keywords:** mycovirus, mitovirus, mitochondrial virus, MtMV1, *Melanconiella theae*, *Camellia sinensis*

## Abstract

A dsRNA segment was identified in the fungus *Melanconiella theae* isolated from tea plants. The complete dsRNA sequence, determined by random cloning together with RACE protocol, is 2,461 bp in length with an AU-rich content (62.37%) and comprises a single ORF of 2,265-nucleotides encoding an RNA-dependent RNA-polymerase (RdRp, 754 amino acids in size). The terminus sequences can fold into predicted stable stem-loop structures. A BLASTX and phylogenetic analysis revealed the dsRNA genome shows similarities with the RdRp sequences of mitoviruses, with the highest identity of 48% with those of grapevine-associated mitovirus 20 and Colletotrichum fructicola mitovirus 1. Our results reveal a novel member, tentatively named Melanconiella theae mitovirus 1 (MtMV1), belongs to the family *Mitoviridae*. MtMV1 is capsidless as examined by transmission electron microscope, efficiently transmitted through conidia as 100 conidium-generated colonies were analyzed, and easily eliminated by hyphal tipping method combined with green-leaf tea powder. MtMV1 has a genomic sequence obviously divergent from those of most members in the family *Mitoviridae* and some unique characteristics unreported in known members. This is the first report of a mycovirus infecting *Melanconiella* fungi to date.

## Introduction

Mycoviruses (also known as fungal viruses) are widespread in all major taxa of fungi (Strauss et al., 2000; Lim et al., 2005; Ro et al., 2007; Stielow et al., 2011; Magae, 2012). Mycoviruses are usually associated with symptomless (cryptic or latent symptoms) infections (Kamaruzzaman et al., 2019). However, some can attenuate the virulence of their fungal hosts and contribute as biocontrol agents for preventing the diseases induced by phytopathogenic fungi (Wu et al., 2007; Ghabrial and Suzuki, 2009; Pearson et al., 2009; Abid et al., 2018; Mizutani et al., 2018). According to the International Committee on Taxonomy of Virus^1^, mycoviruses have been taxonomically grouped into 19 families, including six with dsRNA genomes (*Chrysoviridae*, *Partitiviridae*, *Quadriviridae*, *Reoviridae*, *Totiviridae*, and *Megabirnaviridae*), nine with positive-sense single-stranded RNA (+ ssRNA) (*Alphaflexiviridae*, *Barnaviridae*, *Botourmiaviridae*, *Deltafleiviridae*, *Endornaviridae Gammaflexiviridae*, *Hypoviridae*, *Mitoviridae*, and *Narnaviridae*) (Kamaruzzaman et al., 2019), one with negative-sense ssRNA (-ssRNA; *Mymonaviridae*) (Liu et al., 2014), one with ssDNA (*Genomoviridae*) (Yu et al., 2010), two with reverse-transcribing RNA (*Metaviridae* and *Pseudoviridae*), and unclassified taxa (Liu et al., 2014; He et al., 2018). The number of mycovirus primarily increased in recent years through deep sequencing analysis, and most of the mycoviruses are derived from major phytopathogenic fungi isolated from crops, fruits, and forest trees. In contrast, to date, there are much less reports on mycoviruses associated with fungi isolated from tea plants [*Camellia sinensis* (L.) O. Kuntze], which originated from China and has been cultivated for over 3,000 years.

Mitoviruses (family *Mitoviridae*) represent the simplest group of all RNA viruses (Hillman and Cai, 2013), with a unique genome of + ssRNA which ranges in size between 2.0 and 4.5 kb, encompassing a single long open reading frame (ORF) which encodes a putative RNA-dependent RNA polymerase (RdRp) with six conserved amino acid motifs (I–IV) (Osaki et al., 2015; Ran et al., 2016; Chen et al., 2017; Wang et al., 2019; Kocanová et al., 2020; Liu et al., 2021). Their Genomes are AU rich, especially in the third position of codons (Hong et al., 1998; Xie and Ghabrial, 2012; Liu et al., 2019; Torres-Trenas and Pérez-Artés, 2020; Wang et al., 2021). Mitoviral genomes are characterized by multiple UGA codons encode tryptophan (Trp) instead of stop translation (Nibert, 2017; Nibert et al., 2019); however, the termination codon is usually UAA and sometimes UAG (Nibert, 2017). The terminus sequences could be folded into stable genomic secondary structures that have crucial roles in replicating and protecting their genome (Khalifa and Pearson, 2013; Sahin and Akata, 2019; Torres-Trenas and Pérez-Artés, 2020; Liu et al., 2021). However, mitoviruses are initially found infecting fungi, they have recently been reported from plants as expected infectious entities (Nibert et al., 2018; Nerva et al., 2019; Vong et al., 2019). They are transmitted through horizontal transmission (hyphal anastomosis and heterokaryosis) or vertical transmission (spores) (Romeralo et al., 2018; Kamaruzzaman et al., 2019). Mitoviruses are the only viruses associated with the mitochondria in their hosts (Cole et al., 2000; Osaki et al., 2005; Nibert, 2017; Fonseca et al., 2021) and often led to hypovirulence in their fungal hosts (Nuss, 2005; Xu et al., 2015; Yang et al., 2018).

The genus *Melanconiella* (order Diaporthales, ascomycetes) involves saprophytes and endophytes as well as phytopathogenic fungi causing cankers, diebacks, and rots on crops, ornamentals, and forest trees (Du et al., 2017). Members of the fungus *Melanconiella* were mostly observed to be highly host-specific pathogenic and endophytic fungi of the host family *Betulaceae* especially in the north temperate zone (Voglmayr et al., 2012). To our knowledge, no mycoviruses have been reported from *Melanconiella theae*. Here, we characterized a novel mitovirus from *M. theae* that represent the first mycovirus from *Melanconiella* fungi. It will expand our understanding of the origin, ecology, and evolutionary pathways of mycoviruses (Ghabrial, 1998; Ghabrial and Suzuki, 2009).

## Materials and Methods

### Fungus Isolation and Identification

*Melanconiella theae* strains WJB-5 (Supplementary Figure 1), WJB-1-1, WJB-1-2, WJB-1-9, WJB-1-12, WJB-1-20, WJT-1-1, WJT-1-3, WJT-1-7, WJT-1-9, WJT-1-12, and WJT-1-13 were isolated from naturally infected tea plants showing necrotic symptoms (Supplementary Figure 1A) collected from Xuanen county, Hubei province, China, and identified based on the internal transcribed spacer (ITS) region. Of these strains, WJB-1-9 and WJB-1-12 have the same genetic background with WJT-1-9 and WJT-1-12, respectively, since they were derived from the same strain by culturing single mycelium. Fungal mycelia of these strains were grown on PDA for a week at 25°C and then stored in a 25% (v/v) glycerol solution at −70°C until use.

### Double-Stranded RNA (dsRNA) Extraction

Viral dsRNA was extracted from the fungal mycelial mass using the silica spin column-based method as described previously (Yang et al., 2017). The dsRNAs extracted from *M. theae* strains WJB-1-1, WJB-1-2, WJB-1-9, WJB-1-12, WJB-1-20, and WJB-5, and those from strain WJB-5 were used for viral genome characterization and treated with RNase-free DNase I and S1 Nuclease to remove any DNA and ssRNA remains, fractionated by agarose gel (1%, w/v) electrophoresis, and detected by UV transillumination after staining with ethidium bromide (0.1 mg/mL).

### Complementary DNA (cDNA) Cloning and Sequencing, and RT-PCR Amplication

Purified dsRNA from WJB-5 was used as a template for cDNA synthesis following the previously described method (Xie et al., 2011) using a cDNA synthesis kit (Promega, Madison, WI, United States) and tagged random dN6 primer, 05RACE-3RT (Supplementary Table 1). The resulting cDNA was PCR-amplified using 2 × Gimmico GimiTaq PCR Mix (Gimmico Biotech Co., Wuhan, China) and 05RACE-3 primer (Supplementary Table 1). RT-PCR products were purified and then ligated to the pMD18-T vector (TaKaRa, Beijing, China), then transformed into competent cells of *Escherichia coli* DH5α. Positive clones with cDNA inserts were selected on Luria-Bertani (LB) agar medium containing ampicillin (50 μg/mL), checked with PCR using M13F-47/M13R-48 primers (Supplementary Table 1), and then submitted for sequencing. Specific primers, MtMV1-F and MtMV1-R (Supplementary Table 1) were designed for rapid amplification of cDNA ends (RACE) to amplify terminal sequences of the genome that were not cloned by the initial random cDNA synthesis. For RACE, the PC3-T7 loop adapter (Supplementary Table 1) was ligated to the 3′ end of each strand in the purified dsRNA using T4 RNA ligase (TaKaRa, Beijing, China) at 16°C for 18 h, as described previously (Ran et al., 2016). The oligonucleotide-ligated dsRNA was purified, denatured in DMSO, and subsequently reverse transcribed. RACE and nested PCR amplification were performed using sets of MtMV1-F/PC2 and PC2/MtMV1-R primers (Supplementary Table 1) to amplify the 3′ - and 5′-termini respectively. PCR amplicons were purified, cloned into a pMD18-T vector, and sequenced. All amplicons were sequenced by Tsingke Biological Technology Co., LTD., Wuhan, China. Sequence homology searches were performed with the BLASTX search tool on the National Center for Biotechnology Information, NCBI^2^. Sequence assemblies from the cDNA and the RACE clones were manipulated using DNAMAN software (version 7.0.2; Lynnon Corp., Vaudreuil-Dorion, QC, Canada) to obtain the complete genome sequence of the dsRNA extracted from the strain WJB-5.

RT-PCR amplification was performed using a specific primer pair (MtMV1-F1: 5′-GTCTTATCCGTGTATTCTGGG-3′; MtMV1-R1: 5′-GGCTTGAGGAACATTGAGA-3′), generating a 1981-bp fragment. An annealing temperature of 56°C was used with a PCR Thermal Cycler (Model A200, LongGene, China).

### Phylogenetic and Sequence Analyses

The viral ORF was identified using Expasy^3^ website, and the amino acid (aa) sequences encoded by the ORF were deduced. The yeast mitochondrial genetic code was used for the translation of the ORF. The mycovirus sequence was searched using the NCBI BLASTX program to identify similar mycoviruses for phylogenetic analysis. Maximum-likelihood (ML) phylogenetic tree with 1,000 bootstrap replicates was constructed using DNAMAN software (version 7.0.2; Lynnon Corp., Vaudreuil- Dorion, QC, Canada) for the amino acid sequences of RdRp from WJB-5 dsRNA along with 26 mitoviruses and 4 narnaviruses retrieved from the GenBank database. The evolutionary distances were computed using the Poisson correction method (uniform rates between sites). The potential RNA secondary structures of the 5′- and 3′- terminal sequences were predicted, and the free energy (ΔG) was estimated using CLC genomics workbench software (version 21.0.4; QIAGEN Aarhus, Aarhus, Denmark) as described previously (Liu and Di, 2020). Mitoviral RdRp characteristic motifs were searched using the conserved domain database (CDD) search^4^ provided by NCBI, and multiple amino acid alignments.

### Association of Strain WJB-5 With Viral-Like Particles

Mycelium plugs of the strain WJB-5 were grown on an autoclaved cellophane membrane placed on media for 8 days (d) at 25°C. Minor modifications were adopted to isolate and purify viral particles in the previously described method (Ran et al., 2016). In brief, 35 g mycelia of WJB-5 strain were harvested and crushed in the presence of liquid nitrogen. After that, the crushed mycelia were transferred into new sterile tubes containing 3 volumes of cold phosphate buffer (0.1 M sodium phosphate, pH 7.0, containing 0.2 M KCl and 0.5% β-mercaptoethanol) and gently mixed. The homogenate was centrifuged at 10,000 rpm and 4°C for 15 min to remove the hyphal debris. After three ultracentrifugation cycles, virus particles were purified using a stepwise sucrose gradient of 20–50% (W/V). Following sucrose gradient ultracentrifugation, sucrose fractions were collected, diluted, and subjected to ultracentrifugation to pellet virus particles, and the pellet was resuspended again in 150 μL of phosphate buffer (0.1 mol/L sodium phosphate, pH 7.0). Viral dsRNA was recovered from purified viral particles by a phenol-chloroform-based technique as described previously (Toni et al., 2018), fractionated by agarose gel (1%, w/v) electrophoresis and detected by UV transillumination. For negative staining, 10 μL purified suspension of the virus was loaded on a hydrophobic parafilm surface, and subsequently copper grids with a carbon-formvar coating (200-mesh) were floated onto them for 3 min. After drying with a filter paper, the grids plated with virus particles were immediately refloated for 3 min in a drop of 2% (W/V) phosphotungstic acid solution. The excess suspension was removed using filter paper, and the grids were then air-dried for a few min. Finally, transmission electron microscopy (TEM) was used to visualize the samples.

### Attempts to Cure *Melanconiella* Strains

To eliminate the mycovirus from WJB-5 and five other (WJB-1-1, WJB-1-2, WJB-1-9, WJB-1-12, and WJB-1-20) strains of *Melanconiella theae*, hyphal tipping method together with thermal/dark treatment were conducted. All strains were separately incubated on PDA-T [potato dextrose agar medium containing 0.4% (w/v) green-leaf tea powder] (Yang and Zhang, 2019) media in the dark at 20°C for 3 days. The hyphal tips in the colony margin were individually cut using a fine needle, transferred again to PDA-T in new Petri dishes (1 hyphal tip per dish), and incubated simultaneously. The colonies were subcultured on PDA-T at 20°C in dark conditions for three consecutive subculturing. Finally, the subculturing was achieved on PDA through incubation at 20°C and then used to detect MtMV1 dsRNA. To emphasize that the *M. theae* strains got cured from MtMV1, total RNA was extracted and RT-PCR amplification was performed using specific primers (MtMV1-F1: 5′-GTCTTATCCGTGTATTCTGGG-3′; MtMV1-R1: 5′-GGCTTGAGGAACATTGAGA-3′), as previously described.

### Analysis of Mycovirus Transmission Through Conidia

Conidia of WJB-5 strain (harvested from a 30-d-old culture grown on PDA at 20°C in the dark) were suspended in sterile distilled water to the final concentration of 3 × 10^7^ conidia/mL. The resulting conidial suspension was poured on PDA plates and incubated at 20°C in darkness, and used for culturing of single-conidium isolates. A total of 100 single-conidium isolates derived from WJB-5 were obtained and separately incubated on PDA plates at 20°C for mycelial growth. After the colonies had grown, they were subjected to dsRNA extraction to detect the described virus infection.

### Growth Rate and Morphological Assessments

Morphological traits of virus-infected strains (WJT-1-1, WJT-1-3, WJT-1-7, WJT-1-9, WJT-1-12, and WJT-1-13) and virus-eliminated strains (WJB-5, WJB-1-1, WJB-1-2, WJB-1-9, WJB-1-12, and WJB-1-20) of *M. theae* were assessed as previously described (Jia et al., 2017). Briefly, freshly grown mycelial discs (5 mm in diameter) were transferred onto PDA and incubated in darkness at 25°C. Colony diameter was measured daily for 9 days to calculate growth rates (mm/d).

## Results

### Extraction of dsRNA Segments From *Melanconiella theae* Strains

**FIGURE 1:**
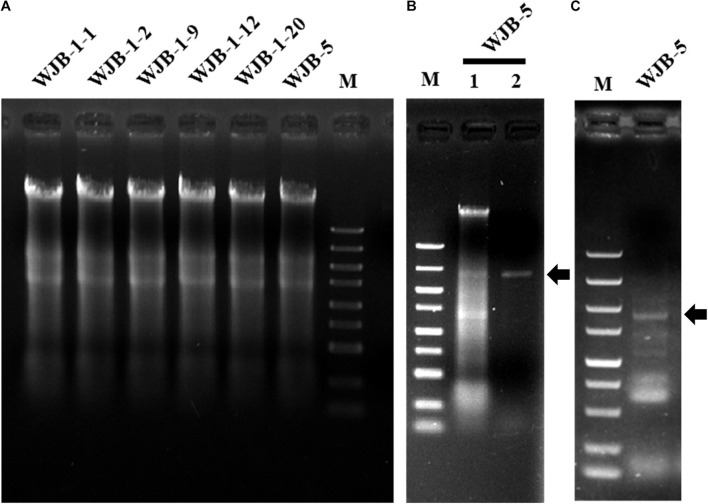
Agarose gel electrophoresis of nucleic acids extracted from *Melanconiella theae* strains, enzyme digest and RT-PCR identification of MtMV1. **(A)** Nucleic acids extracted from strains WJB-1-1, WJB-1-2, WJB-1-9, WJB-1-12, WJB-1-20, and WJB-5. **(B)** The nucleic acids extracted from strain WJB-5 were untreated (Lane 1) or treated with S1 nuclease (Lane 2). **(C)** RT-PCR identification of MtMV1 in strain WJB-5. M: DL5000 DNA marker (100; 250; 500; 750; 1,000; 1,500; 2,000; 3,000; and 5,000 bp). The arrows indicate the target bands.

To check whether *Melanconiella* fungi harbor mycoviruses, six strains (WJB-1-1, WJB-1-2, WJB-1-9, WJB-1-12, WJB-1-20, and WJB-5) from *M. theae* isolated from Hubei province were randomly chosen from the fungal collection and subjected to dsRNA extraction. It revealed that all strains of the phytopathogenic fungus *M. theae* contain mycovirus-like dsRNA segments (Figure 1). After digestion with DNase I and S1 nuclease enzymes, for the strain WJB-5, a clear band of dsRNA approximately 2.40 kb was detected (Figure 1).

### Sequence and Molecular Characterization of WJB-5 dsRNA

The entire cDNA sequence of the dsRNA fragment of the strain WJB-5 was obtained through random priming cDNA synthesis, RT-PCR, and RACE cloning. Sequence assembly of thirty random cDNA clones revealed that the genome was composed of a single dsRNA segment with a molecular size of 2,461 bp and with an AU-rich content of 62.37%. Searching of the full-length genome and the deduced amino acid sequences using BLASTX and BLASTP, the coding protein shared 37.77–48.65% identities with the RdRp sequences of known mitoviruses belonging to the family *Mitoviridae*, and the highest identities are 48.65 and 48.35% with those of grapevine-associated mitovirus 20 (GaMV20) and Colletotrichum fructicola mitovirus 1 (CfMV1), respectively (Supplementary Table 2). Thus, the dsRNA component is proposed as a novel mitovirus and tentatively named Melanconiella theae mitovirus 1 (MtMV1). The complete nucleotide sequence of MtMV1 has been deposited in GenBank with the accession number MW802251.

### Genomic Organization and Phylogenetic Analysis

**FIGURE 2:**
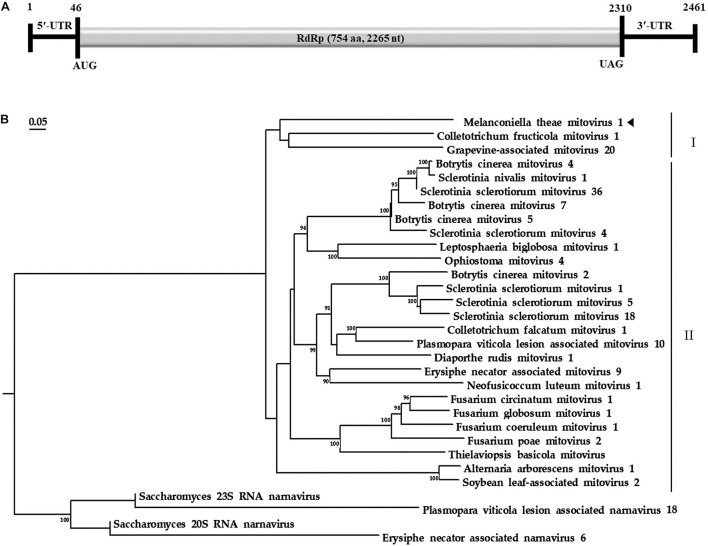
Genomic organization and phylogenetic analysis of MtMV1. **(A)** Genome organization of MtMV1. The start and end positions are labeled for the genome, untranslated regions (UTR), and the open reading frame (ORF). **(B)** ML phylogenetic tree was constructed using DNAMAN software based on RdRp sequences of MtMV1 and other members in the family *Mitoviridae* listed in Table 1. The bootstrap values were deduced based on 1,000 replicates and indicated beside the branches. The evolutionary distances were computed using the Poisson correction method and the branch lengths correspond to the genetic distance; the scale bar refers to the genetic distance of 0.05. A tringle indicates MtMV1.

Based on the yeast mitochondrial code, the genomic organization analysis revealed that the MtMV1 genome contains a single large ORF on the positive sense strand of the dsRNA (Figure 2A).

**FIGURE 3:**
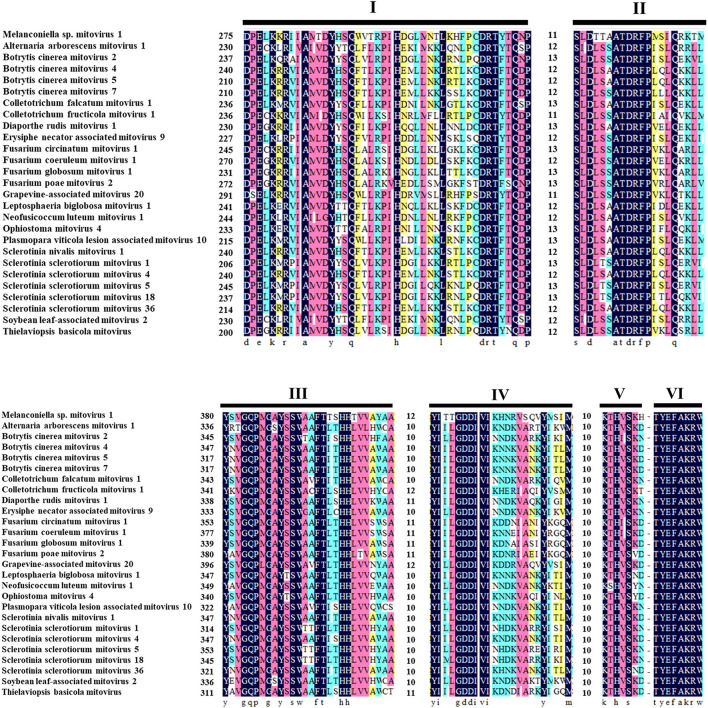
Multiple alignments of the conserved motifs of RdRp in MtMV1 and other mitoviruses. Lines above the aligned sequences indicate to the positions of the motifs (I–VI). Numbers between every two motifs correspond to the number of aa residues separating the motifs. Identical, conserved, and semi-conserved aa residues are color-highlighted with homology level as 100% (black), 75% (magenta), 50% (turquoise), and 33% (yellow).

The ORF starts and terminates at nucleotide positions 46 and 2,310, respectively, of 2,265 bp in size, and potentially codes for an RdRp protein with 754 aa in size. The ORF contains multiple codons (10 UGA and 2 UGG) encoding Trp, and the UAG codon stops the translation. Moreover, the 5′- and 3′-untranslated regions (UTRs) are 45 and 151 bp long, respectively (Table 1). The phylogenetic analysis separated the known mitoviruses into two distinct clusters and placed MtMV1 together with CfMV1 and GaMV20 in cluster I, which is distantly separated from other members (Figure 2B). Based on the conserved domain database (CDD) search and multiple amino acid alignments, the six typical amino acid motifs (I–IV) characterized for the mitoviral RdRp domain superfamily were found to be conserved in MtMV1 (Figure 3). However, MtMV1 contains many unique amino acids as compared with the other members in the conserved motifs (e.g., positions 12 and 20 in motif I, 4, and 19 in motif II, 18 in motif III, 3 and 4 in motif IV), which were composed by threonines (Thr) for MtMV1 while leucines (Leu), isoleucines (Ile) or valines (Val) for the others (Figure 3). Moreover, an unique serine (Ser) in position 16 in motif IV and a unique histidine (His) in position 7 in motif V were observed for MtMV1, while alanine in motif IV and aspartic acids, asparagines or threonines in motif V in these positions for the others (Figure 3).

**TABLE 1:** Comparison of genomic information of MtMV1 and other mitoviruses.

Mitoviruses	Genome (nt)	RdRp (aa)	Tryp codons	Stop codon	5t-UTR size	3′-UTR size	Accession no.
*Melanconiella theae* mitovirus 1	2,461	754	12	UAG	45	151	MW802251
Alternaria arborescens mitovirus 1	2,506	717	10	UAG	218	134	NC_030747.1
*Botrytis cinerea* mitovirus 2	2,496	710	11	UAA	325	40	LN827945.1
*Botrytis cinerea* mitovirus 4	2,768	731	19	UAA	472	100	NC_028474.1
*Botrytis cinerea* mitovirus 5	2,721	731	19	UAA	452	73	MN617167.1
*Botrytis cinerea* mitovirus 7	2,705	731	19	UAA	461	48	MN617168.1
Colletotrichum falcatum mitovirus 1	2,283	703	12	UAA	136	35	MK279482.1
Colletotrichum fructicola mitovirus 1	2,398	709	10	UAA	216	51	LC497424.1
*Diaporthe rudis* mitovirus 1	2,455	701	8	UAA	302	47	MT216310.1
*Erysiphe necator* associated mitovirus 9	2,453	717	11	UAG	232	67	MN557015.1
*Fusarium circinatum* mitovirus 1	2,419	731	13	UAG	156	67	KF803546.1
Fusarium coeruleum mitovirus 1	2,423	757	16	UAG	79	70	NC_026622.1
Fusarium globosum mitovirus 1	2,414	717	15	UAG	188	72	NC_026621.1
Fusarium poae mitovirus 2	2,414	764	13	UAG	65	54	NC_030862.1
Grapevine-associated mitovirus 20	2,467	792	15	UAG	49	39	MW648468.1
*Leptosphaeria biglobosa* mitovirus 1	2,568	756	13	UAA	240	56	NC_040819.1
Neofusicoccum luteum mitovirus 1	2,389	710	9	UAA	128	127	NC_035114.1
Ophiostoma mitovirus 4	2,599	783	13	UAA	204	43	NC_004052.1
*Plasmopara viticola* lesion associated mitovirus 10	2,303	705	10	UAA	182	2*	MN539771.1
Sclerotinia nivalis mitovirus 1	2,720	731	19	UAA	467	57	KT365895.1
*Sclerotinia sclerotiorum* mitovirus 1	2,513	691	12	UAG	418	19	JQ013377.1
*Sclerotinia sclerotiorum* mitovirus 4	2,744	731	19	UAA	464	84	JX401538.1
*Sclerotinia sclerotiorum* mitovirus 5	2,497	718	10	UAG	291	49	KJ462511.1
*Sclerotinia sclerotiorum* mitovirus 18	2,509	722	10	UAG	299	41	KP900925.1
*Sclerotinia sclerotiorum* mitovirus 36	2,732	739	19	UAA	432	80*	MT646380.1
Soybean leaf-associated mitovirus 2	2,477	721	10	UAG	190	121	KT598239.1
Thielaviopsis basicola mitovirus	2,896	705	12	UAG	427	351	DQ173015.2

*Probably incomplete genome.

### Predicted Secondary Structures of the Terminal Sequence

**FIGURE 4:**
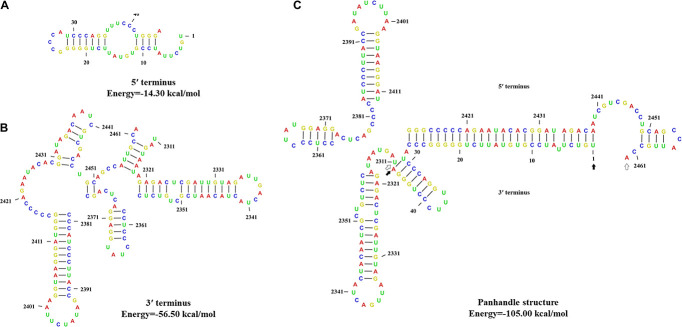
Predicted secondary structures of the terminal sequences of MtMV1. **(A,B)** Stem-loop structures folded for the 5′- and 3′-UTR sequences of the genomic positive-strand, respectively. **(C)** Panhandle structure formed by the reverse complementary regions of the 5′- and 3′-UTRs ends of the MtMV1 genome. The CLC workbench software was used to predict the secondary structures of the terminal sequences and to calculate the free energy. The solid and open arrows indicate the 5′- and 3′-terminal sequences, respectively, in their initial and ending positions.

The 5′- and 3′-terminus sequences of the MtMV1 genome were subjected to the prediction of their secondary structures using the CLC genomic workbench software. The results showed that both 5′- and 3′-UTRs could be folded into potential stem-loop structures that stabilize the RNA genome, with ΔG values of −14.3 and −56.5 kcal/mol, respectively (Figures 4A,B). Additionally, the partial regions of both terminal sequences are reverse complementary, with positions 1 to 27 of 5′-UTR paired with positions 100 to 126 of 3′-UTR, and potentially fold into a panhandle structure with ΔG values of −105 kcal/mol (Figure 4C).

### Attempts to Check the Viral-Like Particles From WJB-5 Strain

To determine whether virions are associated with MtMV1, possible viral proteins were extracted from the mycelia of strain WJB-5 and subjected to ultracentrifugation in stepwise sucrose gradients. The sucrose fraction containing the MtMV1 dsRNAs was further purified and subjected to observation with a transmission electron microscope (TEM). No virus-like particles were observed under TEM, confirming that MtMV1 dsRNAs are naked like other mitoviruses.

### Attempts to Cure WJB-5 Strain From Melanconiella Theae Mitovirus 1

**FIGURE 5:**
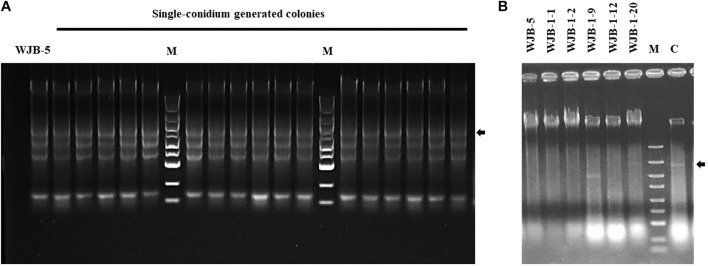
Agarose gel electrophoresis of dsRNA extracted from the mycelia of *Melanconiella theae* strains. **(A)** dsRNA extraction from the mycelia of single-conidium generated colonies derived from strain WJB-5 conidia. **(B)** dsRNA extraction from six strains (WJB-5, WJB-1-1, WJB-1-2, WJB-1-9, WJB-1-12, and WJB-1-20) after being treated with hyphal tipping combined with antiviral extracts. **(C)** Untreated WJB-5 as a control. M: DL5000 DNA marker (100; 250; 500; 750; 1,000; 1,500; 2,000; 3,000; and 5,000 bp). The arrows indicate the target viral dsRNA bands.

To cure the MtMV1 from the WJB-5 strain and the other five strains of *M. theae* (WJB-1-1, WJB-1-2, WJB-1-9, WJB-1-12, and WJB-1-20), the hyphal tipping method combined with antiviral extracts (incorporating green-leaf tea powder in the culture medium) was used. After three sequential rounds of subculturing, MtMV1 was detected by dsRNA extraction. The results showed that MtMV1 was cured from all detected strains (Figure 5B), which was further confirmed by RT-PCR identification resulting in no target bands (1.98 kb in size) (Supplementary Figure 2).

### Vertical Transmission of Melanconiella Theae Mitovirus 1 Through Conidia

To investigate whether MtMV1 can be vertically transmitted to the next generation through fungal conidia, conidial suspension prepared from the strain WJB-5 were smeared on PDA, and a total of 100 single-conidium generated colonies were subjected to dsRNA extraction. The results showed that MtMV1 was retained in all these subisolates, suggesting that MtMV1 has efficiently transmitted through the conidia of *M. theae* (Figure 5A).

### *Melanconiella theae* Mitovirus 1 Confers No Obvious Effects on the Growth Rates and Morphologies of the Host Fungus

To access the effect of MtMV1 on the host fungus, six MtMV1-infected and six MtMV1-free strains of *Melanconiella* spp. were cultured on PDA in darkness to access their growth rates and morphologies. Most of these strains had similar morphologies and growth rates, ranging from 4.4 to 6.1 mm/d for the MtMV1-infected strains and 4.1 to 5.4 mm/d for the MtMV1-free ones, suggesting that no obvious effects were observed for MtMV1 related to the morphologies and growth rates of their host strains (Supplementary Figure 3).

## Discussion

A novel mycovirus, named MtMV1, was identified infecting the phytopathogenic fungus *M. theae*. MtMV1 shares most characteristics of members in the family *Mitoviridae* considering that mitoviruses have the simplest positive ssRNA genomes that harbor a single ORF encoding a putative viral RdRp for their replication (Cole et al., 2000; Hillman and Cai, 2013; Khalifa and Pearson, 2013), capsidless (Hillman and Cai, 2013), and their RdRps contain six conserved amino acid motifs (I–IV) (Osaki et al., 2015; Ran et al., 2016; Chen et al., 2017; Wang et al., 2019; Kocanová et al., 2020; Liu et al., 2021). Consequently, MtMV1 together with GaMV20 and CFMV1 were placed in cluster I as the third member in this group.

Since mitoviruses essentially replicate within mitochondria of their hosts (Polashock and Hillman, 1994; Cole et al., 2000; Osaki et al., 2005; Nibert, 2017; Fonseca et al., 2021), MtMV1 genome shares the most features of mitochondrial codons described before, e.g., the MtMV1 genome contains ten UGA codons, which encode Trp rather than function as a stop codon (Nibert, 2017; Vong et al., 2019), while only two UGG, and has rich of A-U content (62.37%) like other members (59.55 to 73.25%). Moreover, the initiation codon of MtMV1 ORF has a preference to be either A or U with a frequency of 68.3% in the third nucleotide position, similar to those of other members (Liu et al., 2019; Torres-Trenas and Pérez-Artés, 2020; Wang et al., 2021) and has a UAG stop codon, similar to the observation in other members with either UAA or UAG (Nibert, 2017). Additionally, the 5′- and 3′-terminus sequences of MtMV1 could be folded into stem-loop structures, and their reverse complementary regions allow to form a stable panhandle structure, which is also observed in other members (Hong et al., 1999; Khalifa and Pearson, 2013; Wang et al., 2021). Whereas, the size of MtMV1 3′-UTR (151 nt) is relatively longer than its 5′-UTR (45 nt), it is different as compared with other mitoviruses since their 3′-UTR sizes are all shorter than their 5′-UTR (ranging from 1 to 413 nucleotide longer) (Stielow et al., 2011; Xie and Ghabrial, 2012; Nibert et al., 2018). It is worth noting that MtMV1 has the smallest 5′-UTR in size compared with other members (ranging from 49 to 472 nt) in the family *Mitoviridae* whether some biological functions related to the unique traits of MtMV1 terminal sequences require further studies. Moreover, MtMV1 harbors a genomic sequence obviously divergent from those of most members in the family *Mitoviridae* as exemplified by having Thr-rich RdRp motifs as compared with other members (Figure 3).

MtMV1 is efficiently transmitted through all conidia of strain WJB-5 (100 conidium-generated colonies were checked) in agreement with those mitoviruses detected in a high percentage of the individual spore isolates, as exemplified by Fusarium circinatum mitovirus 1 (FcMV1) and Fusarium circinatum mitovirus 2-2 (FcMV2-2) that showed vertical transmission rates between 60 and 100% depending on the fungal isolate (Romeralo et al., 2018), however, both Fusarium verticillioides mitovirus 1 (FvMV1) and Fusarium andiyazi mitovirus 1 strain 162 (FaMV1-162) showed 100% vertical transmission rate through fungal conidia (Jacquat et al., 2020). In contrast, some mycoviruses are less efficiently transmitted through conidia, as exemplified by Colletotrichum camelliae filamentous virus 1 (CcFV-1) and Pestalotiopsis theae chrysovirus-1 (PtCV1) (Jia et al., 2017; Zhou et al., 2021). Generally, the efficient transmission of mitoviruses through asexual spores and sclerotia has hindered to understand mitovirus biology, partly due to lacking mitovirus-free sub-isolates used for infection, although a protoplast fusion-based protocol for horizontal transmission of a mitovirus has been developed (Shahi et al., 2019). In light of several reports of tea polyphenols exhibiting antiviral activities against various viruses, especially positive-sense single-stranded RNA viruses (Okada, 1978; Zhang et al., 2020; Mhatre et al., 2021), we tried the elimination of MtMV1 from the fungal host, it is worth noting that MtMV1 was easily cured by the hyphal tipping method combined with green-leaf tea powder in the culture medium as examined by dsRNA extraction and RT-PCR identification, which is expected to provide an efficient approach to obtain mitovirus-free host strains. Moreover, MtMV1 confers no obvious effects on the growth rates and morphologies as demonstrated by some homologous and isogenous strains. Thus, we did not proceed to viral transmission for more strict assessment of its biological effects.

Collectively, a novel mitovirus (MtMV1) from *M. theae* was characterized based on its taxonomic analysis, molecular characterization, genome organization, electron microscope observation, and some biological traits. MtMV1 harbors a genomic sequence obviously divergent with those of most members in the family *Mitoviridae* and some unique characteristics unreported in known members, contributing helpful information to understand the evolution, molecular and biological traits of mycoviruses.

## Data Availability Statement

The datasets presented in this study can be found in online repositories. The names of the repository/repositories and accession number(s) can be found in the article/Supplementary Material.

## Author Contributions

WX supervised and designed the experiments. KS conducted most of the experiments and wrote the manuscript. MU improved the English and conducted part of experiments. KS and MU analyzed the data. HY and HA conducted initial experiments. MU, HA, ZW, DN, and WX revised the manuscript. All authors read and agreed to the published version of the manuscript.

## Conflict of Interest

The authors declare that the research was conducted in the absence of any commercial or financial relationships that could be construed as a potential conflict of interest.

## Publisher’s Note

All claims expressed in this article are solely those of the authors and do not necessarily represent those of their affiliated organizations, or those of the publisher, the editors and the reviewers. Any product that may be evaluated in this article, or claim that may be made by its manufacturer, is not guaranteed or endorsed by the publisher.

## Footnotes

1
https://talk.ictvonline.org/taxonomy/

2
http://www.ncbi.nlm.nih.gov/

3
https://web.expasy.org/translate/

4
https://www.ncbi.nlm.nih.gov/cdd
