# Corrigendum: Characterization of a novel mitovirus infecting *Melanconiella theae* isolated from tea plants

**DOI:** 10.3389/fmicb.2024.1448885

**Published:** 2024-07-17

**Authors:** Karim Shafik, Muhammad Umer, Huafeng You, Hamdy Aboushedida, Zhenhua Wang, Dejiang Ni, Wenxing Xu

**Affiliations:** ^1^Hubei Hongshan Laboratory, Huazhong Agricultural University, Wuhan, China; ^2^Department of Plant Pathology, Faculty of Agriculture, Alexandria University, Alexandria, Egypt; ^3^Key Laboratory of Horticultural Plant Biology, College of Horticulture and Forestry Sciences, Ministry of Education, Huazhong Agricultural University, Wuhan, China; ^4^Key Lab of Plant Pathology of Hubei Province, Wuhan, China; ^5^College of Plant Science and Technology, Huazhong Agricultural University, Wuhan, China; ^6^Technology Center of Wuhan Customs District, Wuhan, China

**Keywords:** mycovirus, mitovirus, mitochondrial virus, MtMV1, *Melanconiella theae*, *Camellia sinensis*

In the published article, there was an error in affiliation 1. Instead of “Hubei Hongshan Laboratory, Wuhan, China”, it should be “Hubei Hongshan Laboratory, Huazhong Agricultural University, Wuhan, China.”

The authors apologize for this error and state that this does not change the scientific conclusions of the article in any way. The original article has been updated.

