# Supplementary material for: Characterization of a Novel Mitovirus Infecting Melanconiella theae Isolated From Tea Plants
Source: Front Microbiol. 2021 Nov 17;12:757556. doi: 10.3389/fmicb.2021.757556 (PMC8635788; doi:10.3389/fmicb.2021.757556)
Supplement: Supplementary file 1 [file Data_Sheet_1.docx]

**Supplementary Figure 1.** Morphologies of *Melanconiella* sp. and the related symptoms. (A) The necrotic symptoms associated with *Melanconiella* spp. (B) The colony of *Melanconiella* sp. WJB-5 strain was cultured on PDA for 7 days at 25℃.(C and D) Conidial structures and conidia of WJB-5 strain after culture on PDA for 30 days.


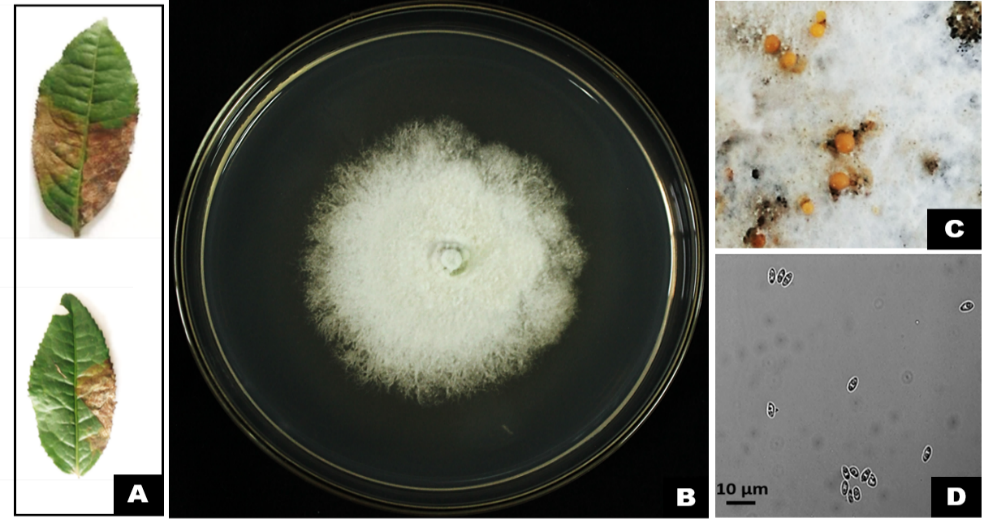


**Supplementary Figure 2.** RT-PCR identification of MtMV1 in cured strains of *Melanconiella theae*. M, marker; C, MeMV1-infected WJB-5 strain. WJB-, cured strains.


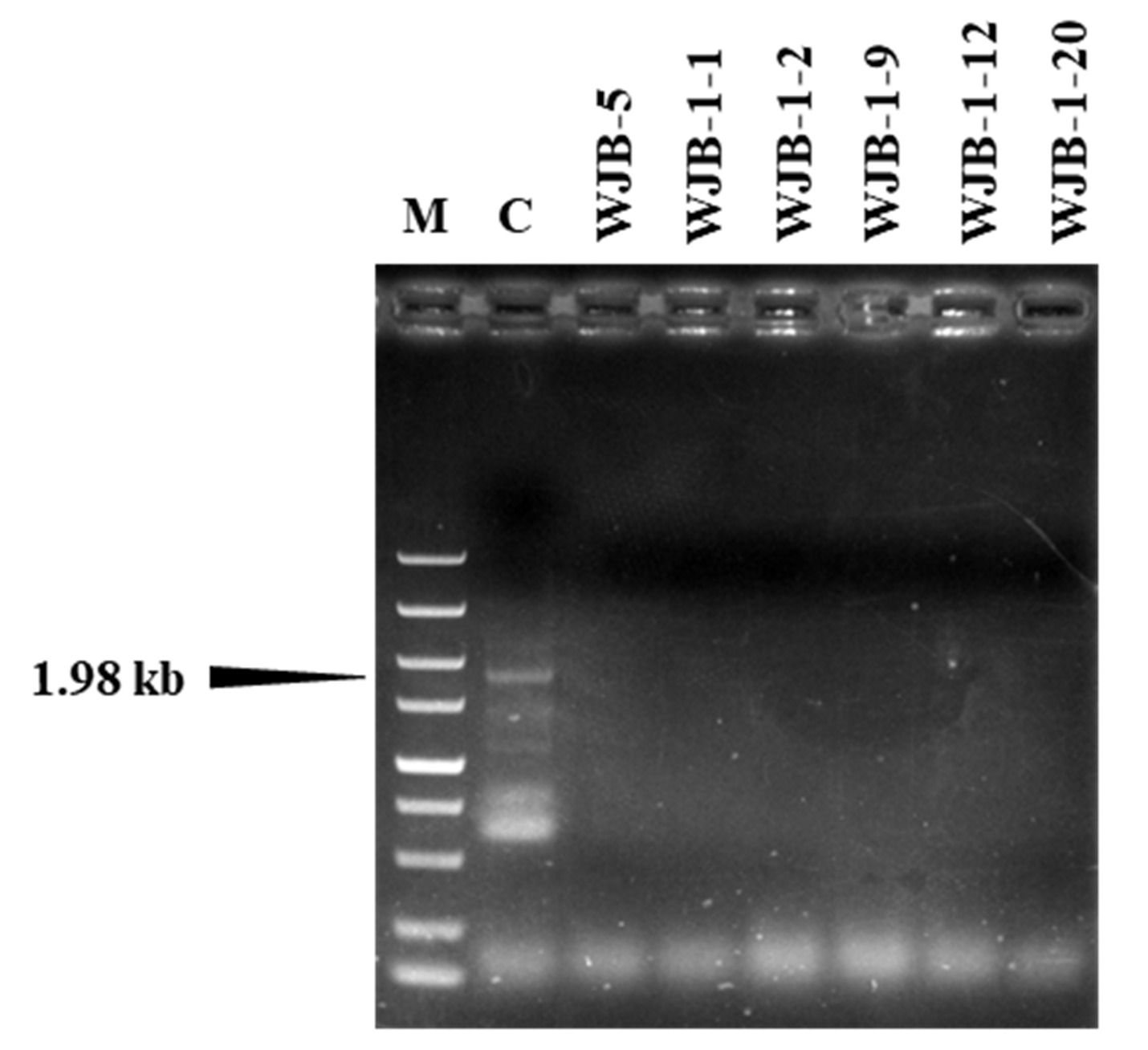


**Supplementary Figure 3.** Morphologies and growth rates of MeMV1-infected and –free strains of Melanconiella spp.. A and B, the colonies (A) of Melanconiella spp. cultured on PDA at 25℃ in darkness for 9 days and the bar graph for their growth rates(B), respectively.


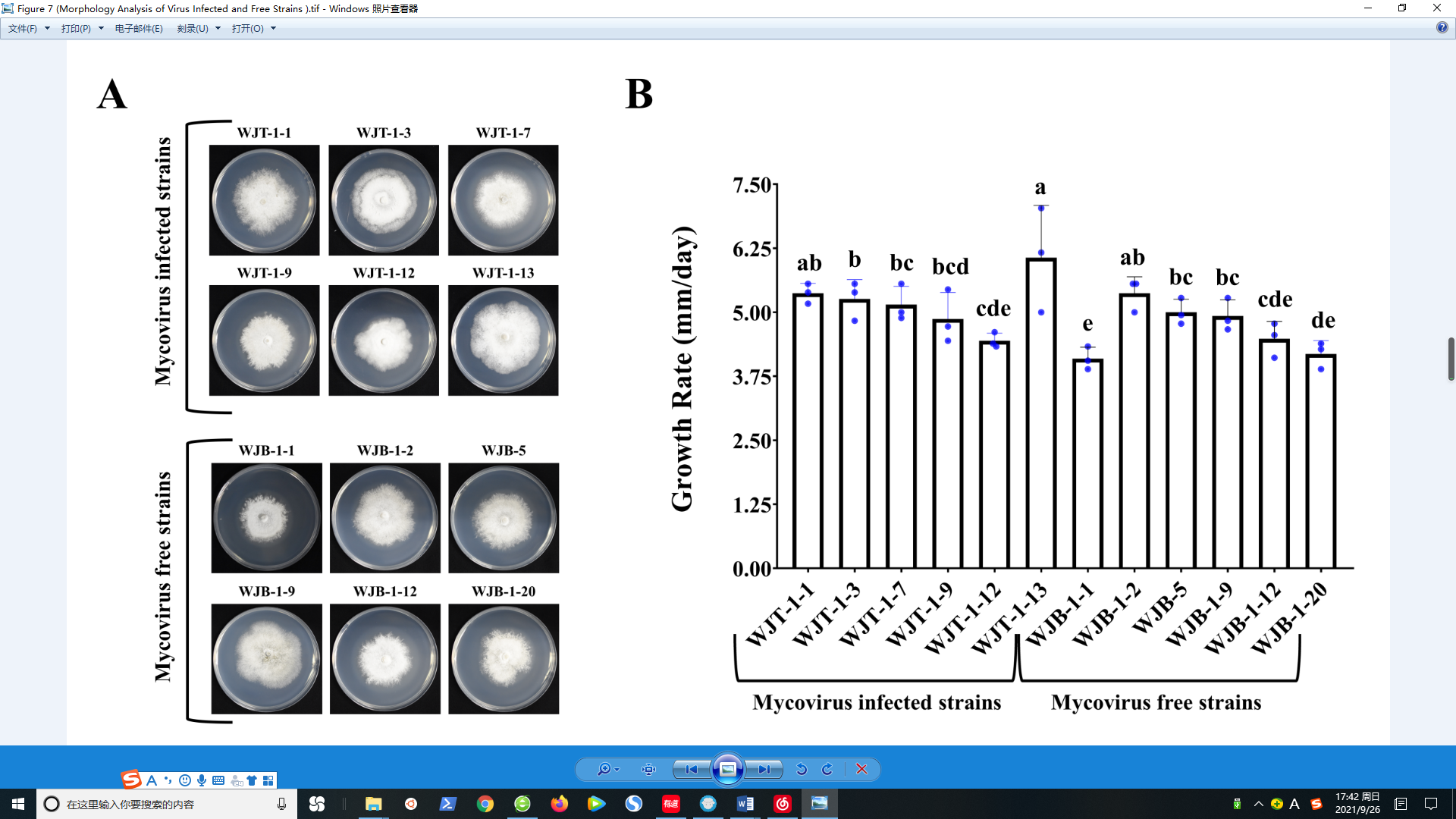


**Supplementary Table 1.**Primers and adapter used in virus amplification.

| Primer/Adaptor | Primer sequence (5'→3') |
| --- | --- |
| 05RACE-3RT | CGATCGATCATGATGCAATGCNNNNNN |
| 05RACE-3 | CGATCGATCATGATGCAATGC |
| PC3-T7-loop adapter | GGATCCCGGGAATTCGGTAATACGACTCACTATATTTTTATAGTGAGTCGTATTA |
| PC2 primer | CCGAATTCCCGGGATCC |
| MeMV1-F | GTGATGGACCAACTGGAA |
| MeMV1-R | CTTGATAGCATAAGCGTAACC |
| M13F-47 | CGCCAGGGTTTTCCCAGTCACGAC |
| M13R-48 | AGCGGATAACAATTTCACACAGGA |

**Supplementary Table 2.** Blastx search information for the putative RNA-dependent RNA polymerase (RdRp) proteins of Melanconiella sp. mitovirus 1 and other mitoviruses.

| Mitovirus species | A+U content (%) | Query Coverage (%) | E-value | Identity (%) | Accession no. |
| --- | --- | --- | --- | --- | --- |
| Melanconiella sp. Mitovirus 1 | 62.37 | - | - | - | MW802251 |
| Alternaria arborescens mitovirus 1 | 70.71 | 74 | 1e-108 | 38.20 | YP_009270635.1 |
| Botrytis cinerea mitovirus 2 | 68.14 | 73 | 2e-115 | 38.44 | CEZ26301.1 |
| Botrytis cinerea mitovirus 4 | 68.31 | 74 | 1e-124 | 41.41 | YP_009182163.1 |
| Botrytis cinerea mitovirus 5 | 69.38 | 69 | 9e-122 | 42.24 | QJT73704.1 |
| Botrytis cinerea mitovirus 7 | 68.28 | 74 | 9e-119 | 40.91 | QJT73705.1 |
| Colletotrichum falcatum mitovirus 1 | 69.55 | 74 | 6e-127 | 40.13 | AZT88621.1 |
| Colletotrichum fructicola mitovirus 1 | 65.30 | 84 | 0.0 | 48.35 | BBN51032.1 |
| Diaportherudis mitovirus 1 | 69.24 | 76 | 1e-118 | 39.39 | QNS28825.1 |
| Erysiphe necator associated mitovirus 9 | 71.42 | 74 | 9e-116 | 41.72 | QHD64820.1 |
| Fusarium circinatum mitovirus 1 | 69.65 | 74 | 2e-111 | 39.49 | AHI43533.1 |
| Fusarium coeruleum mitovirus 1 | 71.48 | 76 | 5e-113 | 38.27 | YP_009126873.1 |
| Fusarium globosum mitovirus 1 | 66.11 | 75 | 3e-109 | 39.05 | YP_009126872.1 |
| Fusarium poae mitovirus 2 | 63.29 | 75 | 3e-110 | 37.77 | YP_009272899.1 |
| Grapevine-associated mitovirus 20 | 59.55 | 86 | 0.0 | 48.65 | QXN75374.1 |
| Leptosphaeria biglobosa mitovirus 1 | 70.01 | 76 | 1e-121 | 40.19 | YP_009553599.1 |
| Neofusicoccum luteum mitovirus 1 | 69.40 | 71 | 1e-109 | 40.60 | YP_009388498.1 |
| Ophiostoma mitovirus 4 | 73.25 | 76 | 5e-125 | 41.81 | NP_660179.1 |
| Plasmoparaviticola lesion associated mitovirus 10 | 69.08* | 70 | 9e-121 | 41.47 | QIR30233.1 |
| Sclerotinia nivalis mitovirus 1 | 67.72 | 74 | 4e-122 | 40.93 | ANJ77669.1 |
| Sclerotinia sclerotiorum mitovirus 1 | 61.71 | 73 | 7e-111 | 39.23 | AEX91878.1 |
| Sclerotinia sclerotiorum mitovirus 4 | 69.60 | 71 | 6e-127 | 42.44 | AGC24233.1 |
| Sclerotinia sclerotiorum mitovirus 5 | 60.87 | 73 | 6e-114 | 39.78 | AHX84132.1 |
| Sclerotinia sclerotiorum mitovirus 18 | 61.96 | 73 | 1e-113 | 40.57 | ALD89135.1 |
| Soybean leaf-associated mitovirus 2 | 71.49 | 74 | 2e-115 | 38.08 | ALM62242.1 |
| Sclerotinia sclerotiorum mitovirus 36 | 68.26* | 74 | 6e-123 | 40.93 | QUE49125.1 |
| Thielaviopsis basicola mitovirus | 67.43 | 69 | 2e-111 | 42.45 | AAZ99833.1 |

* Probably incomplete genome.
